# Young adult with Gorham’s disease presenting in an emergency department: a case report

**DOI:** 10.1186/s13256-021-02993-1

**Published:** 2021-08-17

**Authors:** Markku Grönroos, Ari Palomäki

**Affiliations:** 1 Department of Emergency Medicine, Central Hospital of Kanta-Häme, Ahvenistontie 20, 13530 Hämeenlinna, Finland; 2 Faculty of Medicine and Health Technology, Tampere University, University of Tampere, 33014 Tampere, Finland

**Keywords:** Gorham’s disease, Resorption, Osteolysis, Chylothorax

## Abstract

**Introduction:**

Gorham’s disease is a very rare musculoskeletal disorder characterized by progressive resorption of one or more skeletal bones. Most of the 200 cases reported earlier are diagnosed before the age of 40 years. Due to rarity, the diagnosis of Gorham’s disease in the Emergency Department may be very difficult.

**Case presentation:**

We report a case of Gorham’s disease. A 23-year old Caucasian man presented to the Emergency Department with a significant loss of power and sensation of the lower limbs and lower torso. Clinical examination, computed tomography, and magnetic resonance imaging revealed resorption of the ribs and vertebrae, severe kyphosis, and spinal stenosis in the thoracic area. The patient underwent several surgical procedures, including spondylodesis and decompression, and made a good initial recovery. Biopsy confirmed the diagnosis of Gorham’s disease.

**Conclusion:**

We present a young man with Gorham’s disease visiting the Emergency Department. After the proper diagnosis and treatment, our patient had good outcome, although the etiology of this rare disease is uncertain.

## Introduction

Gorham’s disease is a rare condition of uncertain etiology characterized by progressive resorption of one or more skeletal bones [1, 2]. Although the condition was first reported in 1838 by Jackson and meticulously described in 1955 by Gorham, there are about 200 published cases to date [3–5].

There is no evidence of a genetic, malignant, neuropathic, or infectious component involved in the causation of this disorder. The mechanism of bone resorption remains unclear, although it is characterized by local proliferation of small vascular or lymphatic vessels resulting in progressive destruction and resorption of bone [6, 7]. Gorham’s disease may involve men or women of any age group, although most cases are diagnosed before the age of 40 years. Clinical findings and symptoms vary depending on the affected site [5]. The most serious manifestation, which develops in approximately 17% of patients, is pleural effusion (chylothorax), especially when the disease affects ribs, scapula, or thoracic vertebra, as described by Tie *et al*. [8].

As a part of development of the new specialty of Emergency Medicine in Finland, we are continuously analyzing and developing our clinical environment and practices [9–12]. In this paper, we present a rare case of Gorham’s disease presenting to the Emergency Department (ED) of a secondary care hospital in Finland.

## Case presentation

A 23-year old Caucasian nonsmoking man with no past medical history presented to the ED of Kanta-Häme Central Hospital, Southern Finland, in October 2011 with bilateral numbness of the lower limbs. His walking had deteriorated over a period of 2 months. There was no history of recent trauma or infection, although the patient mentioned hearing an abnormal crack in his left scapular area while lifting a crate in June 2011. There was no family history of note. A symmetrical loss of sensation had risen up to lumbar area, and cold sensation in the lower extremities was absent. Power in the lower limbs was significantly reduced so much so that the patient had trouble rising from bed the day attending hospital.

**Fig. 1:**
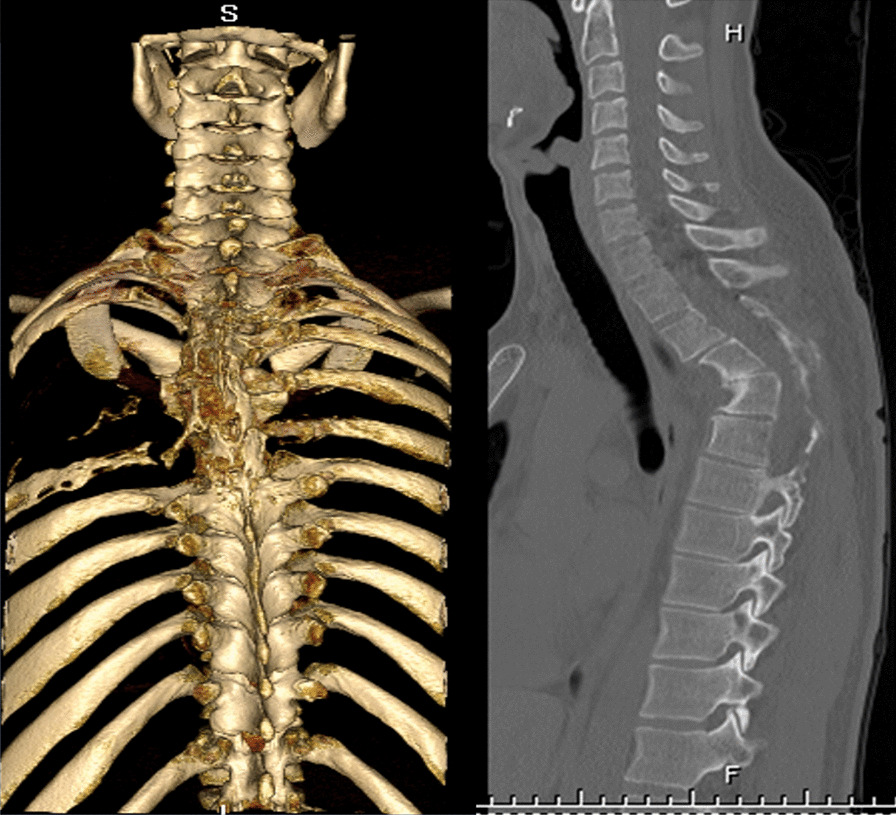
Computed tomography showing destruction of left IV–VI ribs and thoracic vertebrae III–IV together with kyphosis and spinal stenosis of thoracic spine

On admission to ED, clinical examination revealed that the patient walked slowly, and there was evidence of atrophy in the left scapula. Power in the lower limbs, particularly on the left side, was significantly reduced, and atrophy was also visible in the thighs and calves. The patellar reflexes were exaggerated, especially on the left side. A weak extensor response to the plantar reflex (Babinski sign +/+) was found bilaterally. The upper limbs did not show any loss of power or sensory function. Laboratory tests were normal. Analysis of the cerebrospinal fluid showed only a moderate rise in proteins, 773 mg/L. Thoracic magnetic resonance imaging and computed tomography (CT) showed severe kyphosis with severe spinal stenosis as a result of destruction of thoracic vertebrae III–IV and left ribs IV–VI (Fig 1). Radiologically, there were no significant soft-tissue findings.

**Fig. 2:**
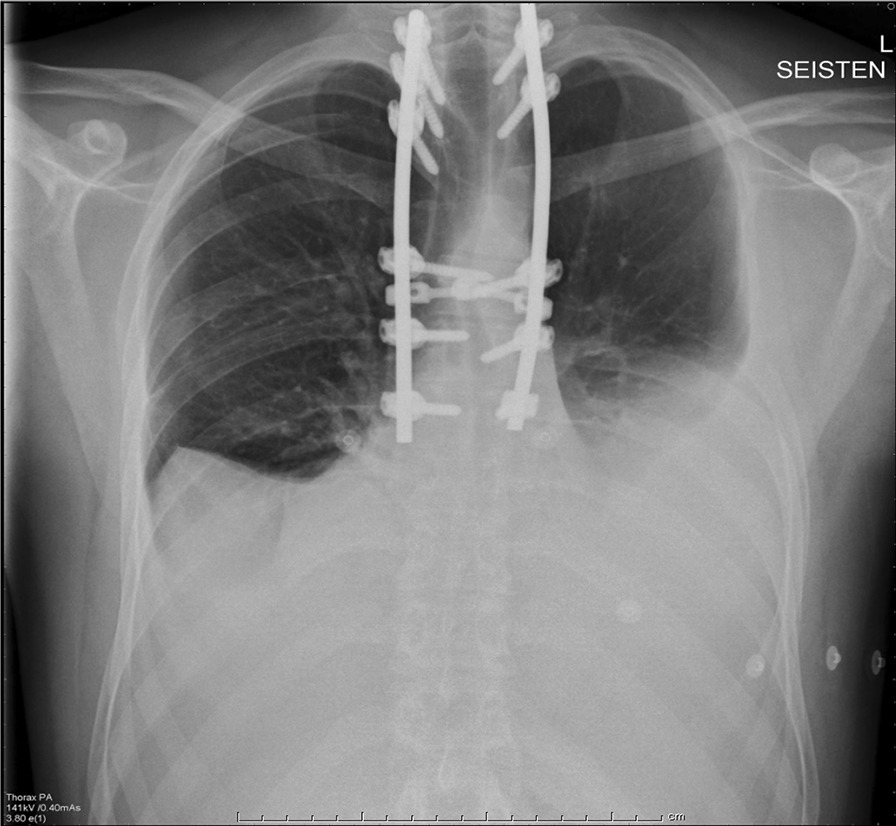
Pleural effusion was revealed on chest x-ray

The patient was hospitalized onto an orthopedic ward, and 2 days later underwent spondylodesis and decompression. Posterior spondylodesis was made bilaterally to thoracic vertebrae II–III and VI–VIII and laterally vertebrae IV with monoaxial and polyaxial screws. Operation proceeded with laminectomy to vertebrae IV–V and lateral decompression of medulla. Kyphosis was corrected with chrome-cobalt rods, and the operation was ended with a bone graft from the patient’s left pelvis. Biopsy confirmed the diagnosis of Gorham’s disease. There were no signs of other diseases that affect vertebrae. Postoperatively, the power in the lower limbs recovered and the patient was able to walk normally. The patient returned home 11 days after the operation. Six weeks postoperatively, the patient developed dyspnea, productive cough, and vomiting, and was found to have bilateral pleural effusions (Fig 2). First pleural aspiration was exudate; analysis showed proteins 48 g/L, lactic dehydrogenase (LD) 128 U/L, leukocytes 7700 × 10^6^/L, and erythrocytes 137,800 × 10^6^/L. Culture showed no bacteria or tuberculosis. Osteolysis in the pelvic area was identified on CT. Frequent pleural aspirations were performed, and the patient developed postpunctional fever. Macroscopically, pleural fluid showed no typical chylothoracic findings. Steroids and antibiotics were started. The patient was then treated with biphosphonates and interferons, which were paused during radiation therapy. Pleural effusion was reaccumulated, and the patient was malnourished, with body mass index (BMI) decrease from 24.6 to 17.1. Albumin was as low as 22 g/L.

Two months after the operation, a thoracic CT showed moderate disease progression in the thoracic vertebrae and profuse pleural effusion. The pleural fluid did not show any pathological or bacterial findings. Biphosphonates, interferons, and radiation therapy were continued. Six months after the operation, pleural effusion proceeded. It was treated with a left chest tube. Pleural fluid was exudate, analysis showed proteins 43 g/L, glucose level was normal, and triglyceride level was negative. Because of re-existing pleural effusion, thoracoabdominal shunt was placed. This time, pleural fluid analysis showed *Propionibacterium acnes*, and the patient was treated with antibiotics. His symptoms and signs, mainly dyspnea, hypoxia, and hypotension, disappeared. Afterwards, the patient was able to walk normally, and fixation in the thoracic spine was appropriate based on imaging.

**Fig. 3:**
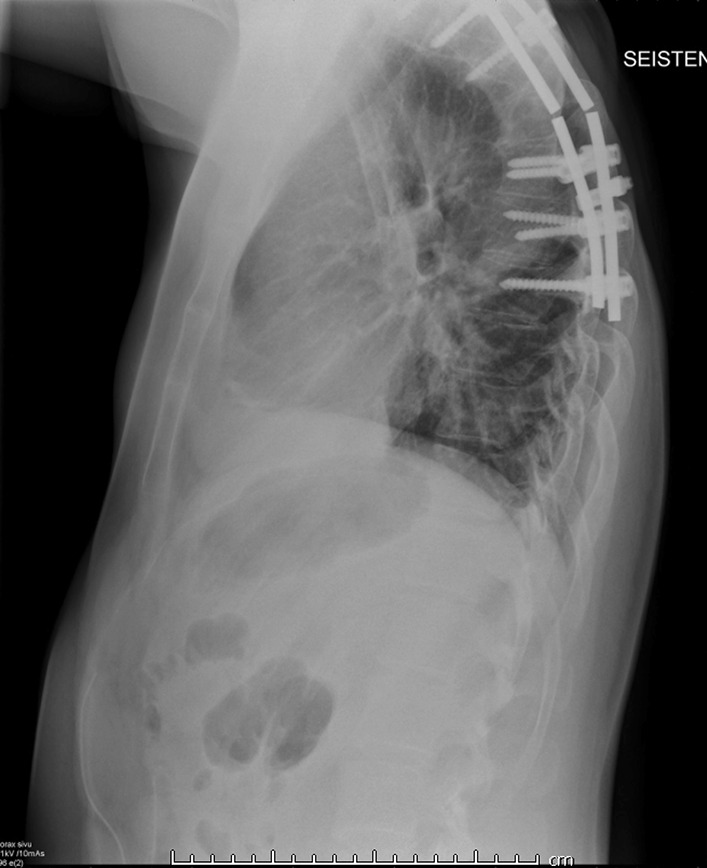
Destruction of the fixation materials on chest x-ray

**Fig. 4:**
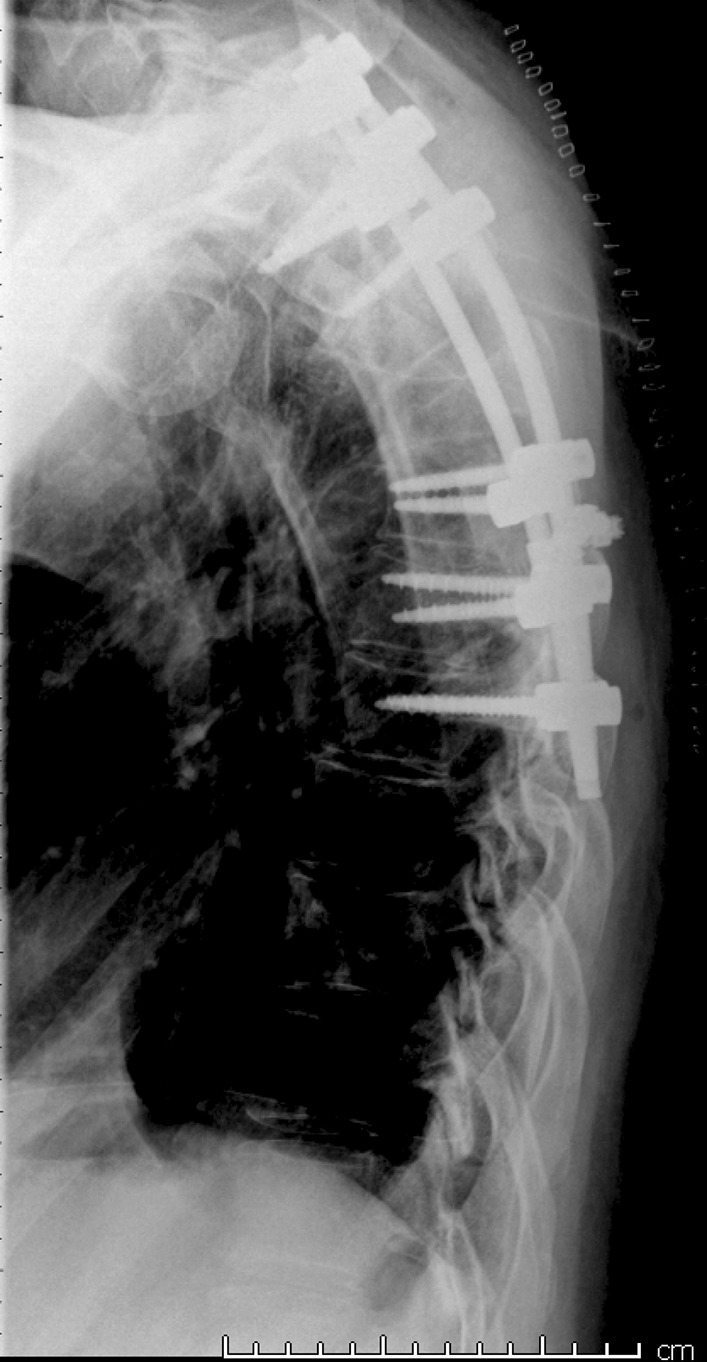
Chest x-ray of postoperative situation

Approximately 3 years after the operation in January 2015, the patient presented to the ED because of back pain after a loud snapping sound in his back. X-ray and CT showed destruction of the fixation materials without worsening of spondylodesis (Fig 3). The patient underwent a surgery with repeat spondylodesis (Fig 4), and there was no evidence of progression of the Gorham’s disease. Since then, he has not visited the ED because of this disease.

## Discussion

In this paper, we have presented a rare case of Gorham’s disease. Radiological investigations identified pathological findings in the thoracic area with resorption of ribs and vertebrae. Following surgical intervention, the patient developed a recognized complication of the disease with pleural effusion, although there were no evidence of chylothorax. This was treated with biphosphonates and interferons. Three years after the diagnosis, the patient underwent further surgery owing to destruction of the fixation material, but there was no evidence of progression of the disease.

Diagnosis of Gorham’s disease is based on clinical, laboratory, radiological, and histopathological findings. Our findings in comparison with published literature were similar with resorption of bones in thoracic area. Tateda *et al*. have reviewed the English literature about Gorham’s disease, and spinal involvement was reported in 59 cases [13]. Disease of the ribs, scapula, or thoracic vertebrae may lead to the development of a chylothorax from direct extension of lymphangiectasia into the pleural cavity or via invasion of the thoracic duct [6]. Tie *et al.* have reviewed reported cases of Gorham’s disease with chylothorax and supported the need of prompt and aggressive surgical intervention in order to improve prognosis [8]. Treatment modalities have not been strictly standardized. Surgery and radiation therapy are the most commonly used, while medical treatments include biphosphonates and interferons.

## Conclusion

Gorham’s disease is a very rare musculoskeletal disorder with variable symptoms depending on the anatomical site affected. Emergency physicians should be aware of patients who present with osteolysis in skeletal bones. Effective therapy is still unknown, and the prognosis of disease is variable. To conclude, we have presented a rare case of a young man with Gorham’s disease with a favorable outcome after proper diagnosis and treatment.

## Data Availability

All data included the informed consent of the patient analyzed during this study are included in this published article.

## Abbreviations

CT: Computed tomography
ED: Emergency Department

## References

[1] Okafuji T, Yabuuchi H, Soeda H, Takahashi N, Hatakenaka M, Sakai S,* et al*. Gorham’s disease of the chest wall: CT and MR characteristics. J Thorac Imaging. 2005;20:284–7. 10.1097/01.rti.0000186997.38447.47 16282906

[2] Radhakrishnan K, Rockson SG. Gorham’s disease: an osseous disease of lymphangiogenesis? Ann N Y Acad Sci. 2008;1131:203–5. 10.1196/annals.1413.022 18519972

[3] Jackson JBS. A boneless arm. Boston Med Surg J. 1838;18:368–9.

[4] Gorham LW, Stout AP. Massive osteolysis (acute spontaneous absorption of bone, phantom bone, disappearing bone). Its relation to hemangiomatosis. J Bone Joint Surg Am. 1955;37:985–1004. 13263344

[5] Tanoue N, Moedano L, Witte M, Montague M, Lukefahr A, Bernas M. Primary versus trauma-induced Gorham-Stout disease. Lymphology. 2018;51:18–27. PMC7852030 30248728

[6] Patel T. Gorham’s disease or massive osteolysis. Clin Med Res. 2005;3:65–74. 10.3121/cmr.3.2.65 PMC1183435 16012123

[7] Hammer F, Kenn W, Wesselmann U, Hofbauer LC, Delling G, Allolio B, Arlt W. Gorham-Stout disease—stabilization during bisphosphonate treatment. J Bone Mineral Res. 2005;20:350–3. 10.1359/JBMR.041113 15647829

[8] Tie ML, Poland GA, Rosenow EC 3rd. Chylothorax in Gorham’s syndrome: a common complication of a rare disease. Chest. 1994;105:208–15. 10.1378/chest.105.1.208 8275732

[9] Lehtonen H, Lukkarinen T, Kämäräinen V, Rautava V-P, Parviainen P, Palomäki A. Improving emergency department capacity efficiency. Signa Vitae. 2016;12:52–7.

[10] Naskali J, Palomäki A, Harjola V-P, Hällberg V, Rautava V-P, Innamaa T. Emergency medicine in Finland: first year experiences of specialist training. Acad J Emerg Med. 2014;13:26–9.

[11] Saarinen HJ, Palomäki A. Acute renal infarction resulting from fibromuscular dysplasia: a case report. J Med Case Rep. 2016;10:118. 10.1186/s13256-016-0895-6 PMC4862219 27159998

[12] Heikkilä I, Kuusisto H, Holmberg M, Palomäki A. Fast protocol for treating acute ischemic stroke by emergency physicians. Ann Emerg Med. 2019;73:105–12. 10.1016/j.annemergmed.2018.07.019 30236416

[13] Tateda S, Aizawa T, Hashimoto K, Kanno H, Ohtsu S, Itoi E, Ozawa H. Successful management of Gorham-Stout disease in the cervical spine by combined conservative and surgical treatments: a case report. Tohoku J Exp Med. 2017;241:249–54. 10.1620/tjem.241.249 28367856

